# Disrupting Hydrogen Bond Network Connectivity With a Double‐Site Additive for Long‐Life Aqueous Zinc Metal Batteries

**DOI:** 10.1002/EXP.20240007

**Published:** 2025-08-21

**Authors:** Dongping Chen, Xipo Ma, Weihao Xu, Chunshuang Yan, Pengbo Lyu, Qiang Zhu, Huaming Yu, Zhenren Gao, Chade Lv

**Affiliations:** ^1^ State Key Laboratory of Space Power‐Sources School of Chemistry and Chemical Engineering Harbin Institute of Technology Harbin P. R. China; ^2^ MIlT Key Laboratory of Critical Materials Technology for New Energy Conversion and Storage School of Chemistry and Chemical Engineering Harbin Institute of Technology Harbin P. R. China; ^3^ Hunan Provincial Key Laboratory of Thin Film Materials and Devices School of Material Sciences and Engineering Xiangtan University Xiangtan P. R. China; ^4^ Institute of Materials Research and Engineering A*STAR Singapore Singapore; ^5^ Hunan Provincial Key Laboratory of Micro‐Nano Energy Materials and Application Technologies College of Physics and Electronic Engineering Hengyang Normal University Hengyang P. R. China

**Keywords:** aqueous zinc‐ion batteries, electrolyte additives, hydrogen bond network

## Abstract

Irregular dendrite growth and complex side reactions pose critical challenges that significantly impede the further industrialization of aqueous zinc‐ion batteries (AZIBs). The “competitive co‐solvents” strategy could introduce hydrogen bond (H‐bond) accepting sites to effectively alleviate the free water molecules. however, it suffers from low conductivity, high cost, and safety risks. Herein, we selected *N*, *N*'‐methylenebisacrylamide (MBA) as a trace additive with amide groups to decrease the activity of water by disrupting the H‐bond. The MBA additive, which incorporates both hydrogen bond donor and acceptor functionalities, successfully restricts H_2_O molecules within a double‐site anchoring configuration. This configuration enhances hydrogen‐bonding interactions and breaks part of the original hydrogen bond network among H_2_O molecules, thereby significantly restraining parasitic side reactions due to the decomposition of active water. Additionally, MBA molecules adsorbed on the surface of the Zn anode could regulate the desolvation and nucleation processes of zinc ions, achieving dense and flat zinc deposition. A high Zn reversibility with Coulombic efficiency (CE) of 99.74% and ultra‐long lifespan of 2800 cycles at 1 and 0.5 mAh cm^−2^ was demonstrated. Besides, a highly reversible Zn electrode significantly boosted the overall performance of Zn//Zn symmetric cells of 1500 h at 5 mA cm^−2^ and Zn//V_2_O_5_ full cell of 2000 cycles at 5 A g^−1^.

## Graphical abstract

Despite the promise of aqueous zinc ion batteries (AZIBs) for safe and sustainable grid storage, water‐induced side reactions at the Zn anode, such as hydrogen evolution reactions (HER), corrosion and dendrite growth, make interfacial water suppression a critical research focus. The incorporated dual‐site MBA additive anchors water molecules via ─C═O and ─NH functional groups, thereby disrupting the original hydrogen bond network among water molecules and significantly suppressing water activity and HER. The proposed strategy reveals the importance of hydrogen bond network regulation for the design of stable aqueous electrolyte, which provides inspiration for electrolyte design for safe, stable, and efficient electrochemical energy storage devices in the future.

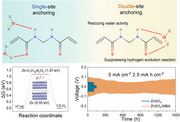

## 1 Introduction

With the rapid development of industry and transportation, the public concern is increasing about the ecological damage and environmental pollution triggered by the grand consumption of fossil fuels [1]. There is a pressing need to develop large‐scale energy storage systems with the merits of economic benefit, high security, high performance, and sustainability [2]. The reliance on combustible and highly toxic organic electrolytes in mainstream lithium‐ion batteries arouses serious safety risks, which hinders the widespread application at the grid level [3]. Getting rid of combustible organic electrolytes with non‐combustible aqueous electrolytes is an achievable strategy to alleviate the safety issues [4]. The incorporation of aqueous electrolytes brings significant benefits, notably by eliminating the need for costly, flammable, and toxic organic electrolytes. Additionally, owing to their inherently low viscosity, aqueous electrolytes exhibit superior conductivity compared to their organic electrolytes [5]. Aqueous zinc‐ion batteries (AZIBs) have emerged as the commercially promising next‐generation energy storage technology due to their inherent safety and eco‐friendliness [6]. Thanks to its high theoretical specific capacity (820 mAh g^−1^), intrinsic safety, and abundant reserves, zinc metal can be directly used as an electrode material in aqueous electrolytes [7]. As the zinc anode possesses a low redox potential (−0.762 V vs. standard hydrogen electrode), however, the competitive hydrogen evolution reaction (HER) process inevitably occurs on the anode surface in the aqueous environment. Along with the adverse HER competition, the local pH value on the surface of the Zn electrode increases, which motivates the formation of passivation by‐products [8]. The loosely stacked corrosion products lead to the surface roughness and heterogeneity of the Zn anode, thereby aggravating the uneven deposition of Zn^2+^. The Coulombic efficiency (CE) continuously decreases owing to the HER and dendritic growth, which further leads to safety issues and battery failure, such as internal short circuits, battery swelling, and even explosion caused by thermal runaway [9]. To tackle with that, it is desirable to reduce the possibility of side reactions and stabilize the Zn anode by inhibiting the activity of water molecules [10].

Recent relevant studies have employed high‐concentration electrolytes or introduced cosolvents to decrease the free water content and interrupt the original solvated balance for suppressing HER [11]. Nevertheless, the high‐concentration salts or combustible organic components may lead to sluggish reaction kinetics and safety issues, accompanied by increased manufacturing costs, which is contrary to the original intent of developing AZIBs [12]. Regulation of the hydrogen bond (H‐bond) between H_2_O molecules in the electrolyte to confine water molecules is another promising strategy for decreasing the activity of H_2_O molecules. Because H_2_O molecules are capable of serving as both hydrogen bond donor (HBD) and hydrogen bond acceptor (HBA), a vast array of H_2_O molecules are intricately linked through H‐bond in aqueous electrolytes to create an extensive hydrogen bond network [13]. In line with the Grotthuss diffusion mechanism, protons are transported to the anode surface by the jumping mechanism [14]. Propelled by the presence of hydrogen bond network and continuous breaking/forming of H─O covalent bonds, the water will be decomposed and the zinc anode will be passivated. Adjusting the number and order of hydrogen bond networks among H_2_O molecules in aqueous electrolytes is essential for suppressing hydrolysis. From this point of view, chemicals that contain several HBA functional groups including ─S═O, ─C≡N, or ─C═O, such as tetramethylene sulfone (TMS) [15], succinonitrile (SCN) [16] and 1,3‐dimethyl‐2‐imidazolidone (DMI) [10a], could serve as additives to regulate the aqueous electrolytes. These HBA‐type additives can effectively break the original hydrogen bond network between H_2_O molecules, thereby substantially reducing the activity of H_2_O molecules. However, it is unrealistic to fully break the hydrogen bond network with additives as a single HBD or HBA. Strategic incorporation of an additive possessing both HBA and HBD functional groups is a feasible approach to effectively anchor H_2_O molecules and regulate the constitution of H‐bonds in aqueous electrolyte.

Herein, we employed *N*, *N*'‐methylenebisacrylamide (MBA) as an additive, which owns carbonyl oxygen and amide protons as HBA and HBD, respectively, to modulate the hydrogen bond network of H_2_O molecules in the aqueous electrolyte for AZIBs. A range of spectroscopic characterization and theoretical calculations indicated that the introduced dual‐site MBA could form hydrogen bonds with H_2_O molecules through simultaneously anchoring ─C═O and ─NH functional groups. This interaction leads to the creation of robust hydrogen bonds between MBA and H_2_O, which contributes to destroying the original hydrogen bond connections among water molecules, significantly reducing the activity of H_2_O molecules and the occurrence of HER. Furthermore, the excellent zincophilicity endowed MBA additive with favorable adsorption onto zinc anode surface to generate a hydrophobic electric double layer (EDL), which could effectively minimize the interaction between H_2_O molecules with the Zn anode, immunize the dendrite formation and promote the interfacial reaction kinetics. Attributed to these merits, the asymmetric cell using such electrolyte achieved a stable lifespan of 2800 cycles with a high CE of 99.74% at 1 mA cm^−2^ and 0.5 mA h cm^−2^. Besides, the symmetric cells in MBA‐contained electrolyte delivered significantly enhanced cycle stability, in terms of an ultra‐long lifespan of 1500 h at 5 mA cm^−2^ and 2.5 mAh cm^−2^. With such elegant additive, the Zn//V_2_O_5_ full cell also displayed high capacity reversibility and long lifespan, highlighting its potential for practical application. This work elucidated the fundamental understanding of regulating the hydrogen bond network connectivity, which offers guidance for designing achievable electrolytes towards stable AZIBs.

## 2 Results and Discussion

### 2.1 Physicochemical Properties of ZnSO_4_/MBA Electrolyte and Hydrogen Bond Network Reconstruction

**FIGURE 1:**
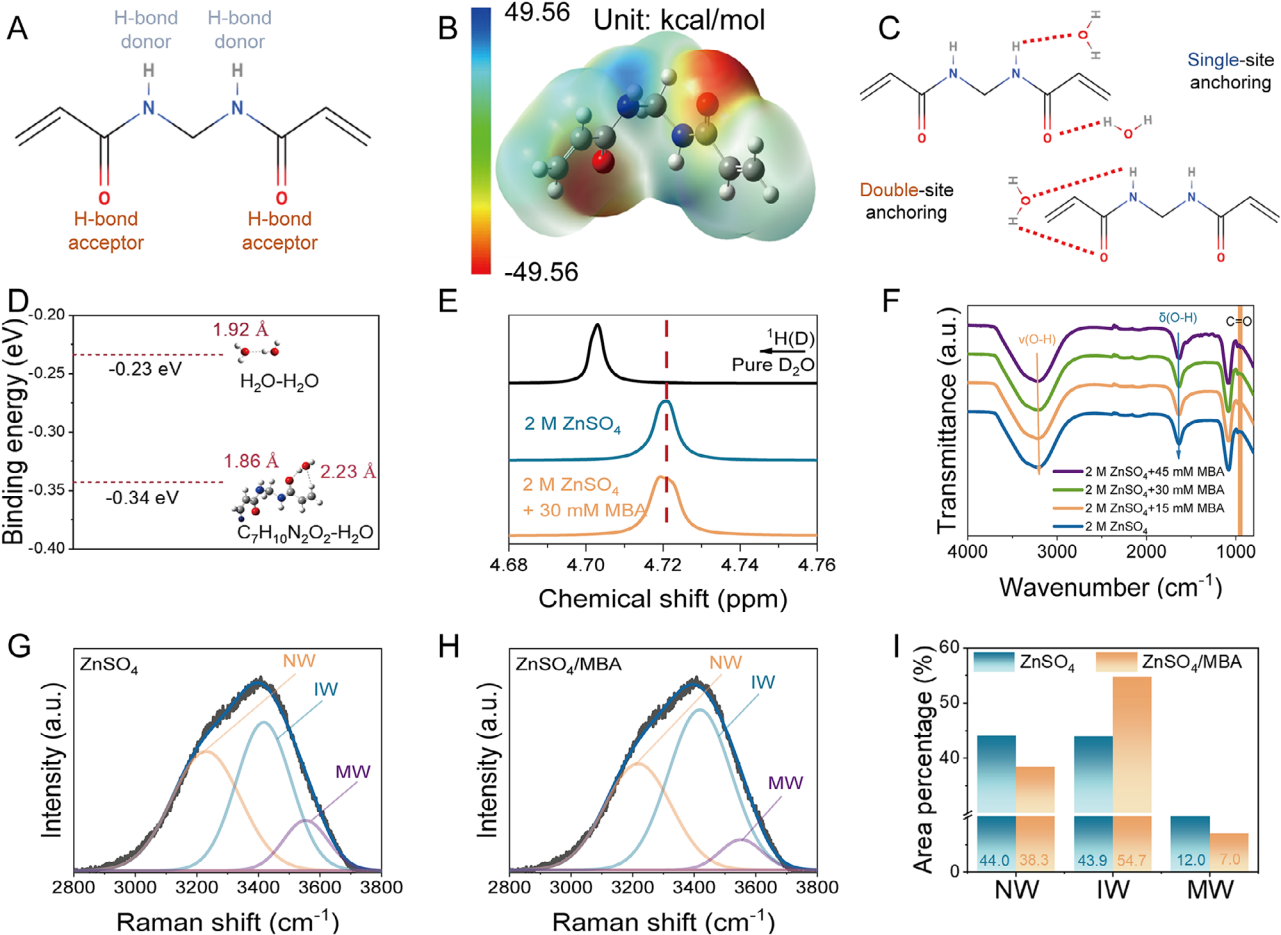
(A) Molecular structure and (B) electrostatic potential of the MBA. (C) The structure of MBA molecule anchored water molecules. (D) The calculated binding energy of H_2_O–H_2_O and MBA‐H_2_O. (E) NMR and (F) FTIR spectra of various electrolytes. Gaussian fitted curves represent three states of water molecules in (G) blank ZnSO_4_ electrolyte, (H) ZnSO_4_/MBA electrolyte, and (I) corresponding fitting results.

The molecular structure and geometry structure of MBA are shown in Figure 1A, with symmetrically distributed amide and carbonyl groups within the molecule. Generally, carbonyl groups with the lone pair electrons on oxygen atom can act as HBA, while ─NH functional groups can donate one hydrogen as HBD [4a]. It is reasonable to believe that the original water hydrogen bond network will be disrupted with HBA and HBD, resulting in a reduction in the activity of water. In addition, the electrostatic potential (ESP) distribution was calculated. It clearly demonstrates that the O atoms within the ─C═O own the highest electronegativity and the primary concentration of electron density (Figure 1B), which offers two robust active sites to Zn^2+^ and water molecules. Because both the ─NH and ─C═O functional groups can participate in forming stronger H‐bonds with water molecules [17], the MBA additive presents two possible anchoring structures (Figure 1C). The initial anchoring structure, known as “single‐site anchoring,” involves a single binding site (either an ─NH or a ─C═O group) from MBA engaging in hydrogen‐bonding interactions with individual water molecules. The “double‐site anchoring” is also enabled by the MBA molecule, of which the both ─NH and ─C═O groups can form H‐bonding with one H_2_O molecule. Furthermore, density functional theory (DFT) calculations were conducted to understand the exact anchoring structure. As illustrated in Figure 1D, the calculated binding energy of MBA‐H_2_O (−0.34 eV) is much lower than that of H_2_O–H_2_O (−0.23 eV), indicating the advantages for MBA to interaction with water molecules via hydrogen bonds. Nuclear magnetic resonance spectroscopy (NMR), Fourier transformed infrared spectroscopy (FTIR) and Raman spectroscopy were used to evaluate the effect of MBA additive. The ^1^H NMR signal from H_2_O occurs a upfield shift after the addition of MBA (Figure 1E). This proves a rise of electronic density ascribed to the strong H‐bonding interactions between MBA and water (C═O···H─O) [18]. Indicated by the FTIR in Figure 1F, the H‐bond number is decreased between H_2_O molecules with disrupted H‐bonding networks as reflected by the res‐shifted O─H stretching vibrations in ZnSO_4_/MBA electrolyte. Figure 1G, H show the Raman spectra of electrolytes with/without MBA additive. The extensive O─H stretching band can be attributed to three distinct states of water molecules, characterized by using a Gaussian function: (1) “network water (NW),” which is linked to water molecules with robust hydrogen bond coordination; (2) “intermediate water (IW)”, corresponding to water molecules which possess irregular hydrogen bonds and are partially connected to other water molecules, yet are insufficient to establish a complete interconnected network; (3) “multimer water (MW),” which is ascribed to water molecules existing as isolated monomers, dimers, or trimers, and exhibits minimal interactions with other electrolyte components [12, 19]. Noticeably, a pronounced reduction in the NW fraction can be observed, with its percentage falling from 54.0% to 38.3%, demonstrating that the robust hydrogen‐bonding network is devastated due to the incorporation of MBA (Figure 1I).When serving as the additive in electrolyte, MBA molecules could form strong hydrogen bonds with water in the system with the increased IW fraction to 20.99%, which is beneficial for the de‐solvation process of Zn^2+^ ions and the inhibition of parasitic reactions stemmed from water decomposition. Therefore, the double‐site anchoring effect endows the MBA additive with the functions as both the hydrogen‐bond donor and acceptor, which significantly amplifies the hydrogen‐bonding interactions with water molecules. The strengthened bonding interactions enable the disruption of hydrogen‐bonding networks among water molecules, effectively suppressing the HER.

In addition to chemical characteristics, the impact of MBA concentration on the reversibility of Zn metal anode was also examined. Specifically, different amounts of MBA were added into the pure ZnSO_4_ electrolyte, the Zn//Cu asymmetric cells and Zn//Zn symmetric cells were further assembled to evaluate the long‐term cycling performance of Zn metal anodes with various electrolytes (Figures S1 and S2). It can be found that the ZnSO_4_ electrolyte with 30 mm MBA additive electrolyte exhibits the longest cycling lifespan and the highest CE compared to other concentrations. Thus, it is determined that the ideal MBA concentration is 30 mm and the corresponding electrolyte is named as ZnSO_4_/MBA.

### 2.2 The Strong Adsorption of MBA and Its Inhibition of Hydrogen Evolution Reaction

**FIGURE 2:**
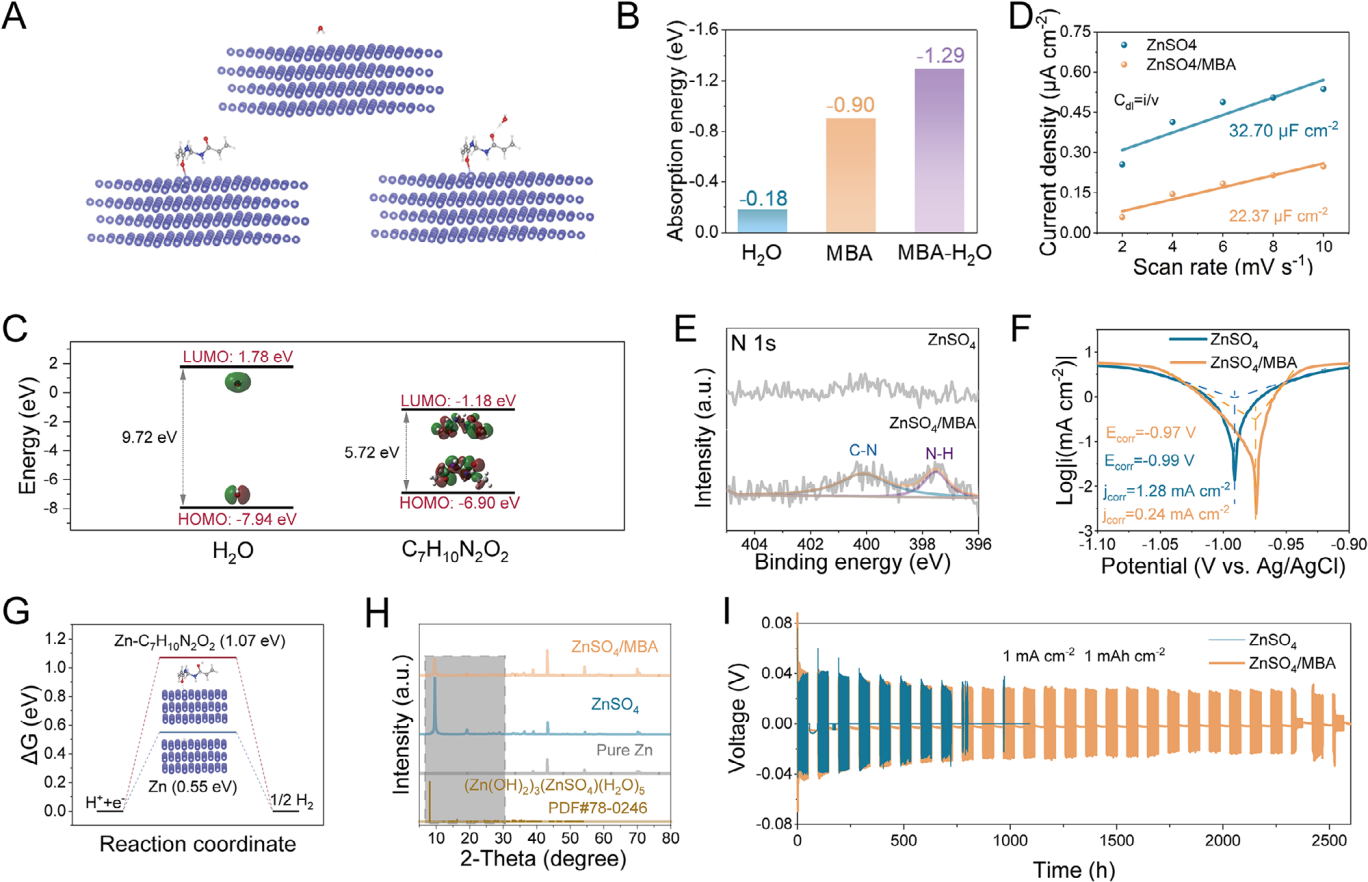
(A) The optimized adsorption model of different molecules on the Zn (002) crystal plane and (B) corresponding calculated absorption energy. (C) The calculated HOMO and LUMO values of H_2_O and MBA. (D) EDLC of symmetric cell in ZnSO_4_ and ZnSO_4_/MBA electrolytes. (E) N 1s XPS spectra of the zinc electrode after 50 cycles using ZnSO_4_ and ZnSO_4_/MBA electrolyte. (F) Tafel plots in two distinct electrolytes. (G) The hydrogen barriers in electrolytes without/with MBA. (H) XRD patterns of fresh zinc foil and zinc anode after cycling in various electrolytes. (I) The shelving and recovery performance of symmetric cells with different electrolytes.

It has been demonstrated that the interaction between MBA and H_2_O through the evolution of H‐bonds in the ZnSO_4_/MBA electrolyte. Notably, due to the trace amount of MBA addition, the strong adsorption ability of the MBA molecules on Zn anodes is the key to take the full advantages of MBA additive. First, the adsorption ability of various molecules on the Zn (002) crystal plane was calculated. The optimized DFT model structure of adsorption energy is displayed in Figure 2A. The absorption energy of MBA molecule (−0.90 eV) on the surface of zinc anode is much lower than that of H_2_O (−0.18 eV) on Zn surface (Figure 2B), suggesting the stronger interaction between MBA and Zn attributable to the polar functional groups in MBA which possess a significant number of unpaired electrons. After the introduction of MBA, the water molecules on the interface tend to be captured by the adsorbed MBA molecules with a calculated value of −1.29 eV, indicating a strong interaction force between the MBA molecules adsorbed on the interface and the water molecules [20].

Furthermore, based on molecular orbital theory, a reduced gap between the lowest unoccupied molecular orbital (LUMO) and the highest occupied molecular orbital (HOMO) energy levels, coupled with a lower LUMO level, signifies an improved capability for electron transfer [21]. This promotes the adsorption of solvent molecules onto Zn metal anodes. Consequently, calculations at the molecular orbital level were conducted to delve deeper into the zincophilic nature of the MBA molecule. As descripted in Figure 2C, the gap between energy levels of the HOMO and LUMO of MBA is much smaller than that of H_2_O. Moreover, the much lower LUMO level of MBA (−1.88 eV) compared to H_2_O (1.78 eV) reveals that the superior adsorption affinity of MBA toward Zn anode [22]. The above theoretical calculation results indicate that MBA prefers to attach to the Zn electrode and captures active water molecules through building strong hydrogen bonds with H_2_O molecules. This would dramatically inhibit the water molecules activity and improve the electrode/electrolyte interface (EEI) stability. The effect of MBA in modifying the anode/electrolyte interface (AEI) is further investigated by cyclic voltammetry (CV) tests, and the current densities are plotted against the scan rate to calculate the EDL capacitance (EDLC). In contrast with pristine ZnSO_4_ electrolyte possessing an EDLC value of 32.70 µF cm^−2^, it declines to 22.37 µF cm^−2^ in ZnSO_4_/MBA electrolyte, which is mainly due to the preferable adsorption of MBA molecules instead of H_2_O molecules on the Zn anode surface (Figure 2D and Figure S3) [20, 23]. The larger size of the MBA molecule can cause an expansion of the stern layer distance and a corresponding reduction in the EDLC. To further prove the above analysis, the cycled Zn anodes with various electrolytes were collected to study the adsorption behavior of MBA through the X‐ray photoelectron spectroscopy (XPS) measurement (Figure 2E and Figure S4). The strong characteristic peaks (C─N and N─H) corresponding to MBA can be found in the high‐resolution C 1s spectra of Zn anode cycling in ZnSO_4_/MBA electrolyte, showcasing the stable adsorptive interaction of MBA with the anode surface throughout the discharge/charge process.

The instrumental role of the MBA additive for inhibiting side reactions was also studied and examined. Initially, zinc foils were soaked in electrolytes with/without MBA for 7 days. The comparison of optical photographs and scanning electron microscope (SEM) images of soaked Zn electrodes are displayed in Figures S5 and S6. In the pure ZnSO_4_ electrolyte, the metallic luster of zinc foil becomes invisible and the Zn foil displays a rough surface with massive flake‐like structures after soaking for 7 days. Furthermore, the formed by‐products on Zn surface are confirmed by X‐ray diffraction (XRD) analysis as (Zn(OH)_2_)_3_(ZnSO_4_)(H_2_O)_5_ (PDF#78‐0426) [24], as shown in Figure S7. The accumulation of by‐products originates from the water‐splitting reaction on the Zn surface, leading to an increased OH^−^ concentration on the anode surface and the consumption of active substances. On the contrary, the Zn foil soaked in ZnSO_4_/MBA electrolyte maintains the smooth surface with the absence of by‐products, manifesting the effective inhibition of chemical corrosion by MBA. As illustrated by the Tafel plots in Figure 2F, the Zn metal anode in the MBA‐contained electrolyte displays an elevated corrosion potential (*E*_corr_) at −0.97 V and a reduced corrosion current density (*I*_corr_) of 0.24 mA cm^−2^, contrasting with the values observed in the pure ZnSO_4_ electrolyte, which are −0.99 V and 1.28 mA cm^−2^, respectively. Besides, a lower exchange current density of Zn electrode and increased oxygen evolution reaction potential can be observed than these in electrolyte without MBA, implying the larger resistance of the HER and the ability to enhance electrolyte stability (Figures S8 and S9). Theoretically, the Gibbs free energies of H^+^ adsorption (Δ*G*) on the zinc anode with different electrolytes were computed to elucidate the mechanism for the suppression of the HER [25], as depicted in Figure 2G. Evidently, a much higher H_2_ formation barrier (Δ*G* = 1.07 eV) occurs on the anode surface in ZnSO_4_/MBA electrolyte, which is higher than that of the blank electrolyte (0.55 eV). This represents a weak HER activity for ZnSO_4_/MBA electrolyte, which fully benefits from the presence of both HBD and HBA in the MBA additive. Ascribed to the strong corrosion inhibition and side reaction suppression of MBA, nearly no corrosion by‐products can be found on the Zn electrode after cycling in MBA‐contained electrolyte (Figure 2H). Furthermore, the shelving and restoring ability of Zn//Zn symmetric cells using electrolyte with/without MBA was examined by intermittent cycling and resting test. The Zn//Zn cell with MBA always restarts normally and performs steadily without apparent voltage fluctuation (Figure 2I). However, the symmetric cell with pure ZnSO_4_ is difficult to withstand such test conditions and fails soon after 750 h. Taken together, through weakening the H─O bond strength among H_2_O molecules with MBA, the MBA adsorption layer conduces to blocking the side reactions and reducing active water in AEI for increasing the stability of Zn anode.

### 2.3 Enhanced Dynamics to Modulate Zn Deposition Behavior

**FIGURE 3:**
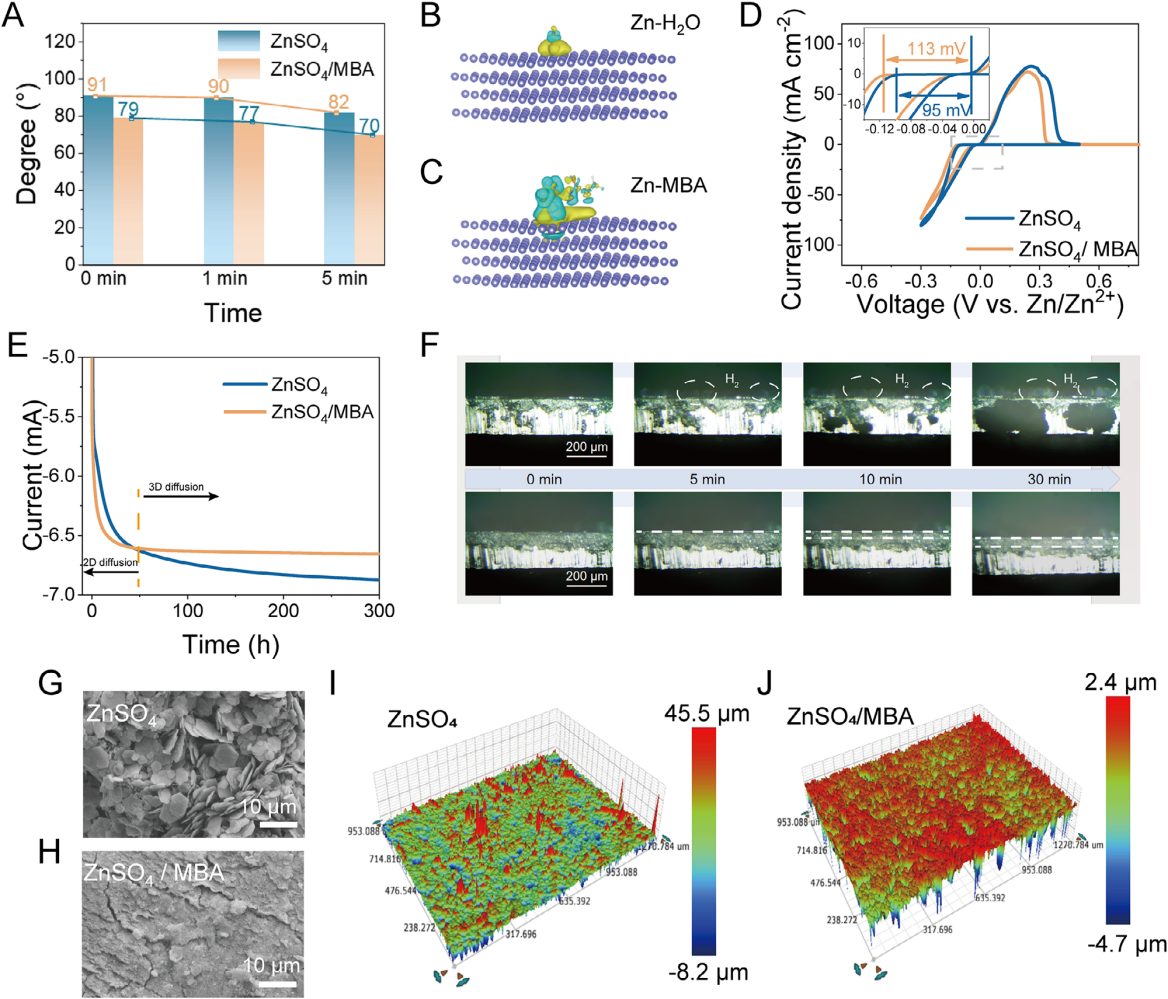
(A) The results of the contact angle variation over time on the surface of Zn foils in different electrolytes. The charge density difference on the Zn foil with (B) H_2_O and (C) MBA molecules adsorbed (yellow and cyan clusters indicate regions of electron density accumulation and depletion, respectively). (D) CV curves of Zn//Ti half‐cell without/with MBA additive. (E) Cyclic voltammetry curves of Zn//Zn cells in various electrolytes. (F) The in situ optical tests of the Zn deposition process using different electrolytes. SEM images of Zn anode surface after Zn deposition in electrolyte (G) without and (H) with MBA. Optical surface‐profilometry images of the Zn electrode after cycling in (I) ZnSO_4_ and (J) ZnSO_4_/MBA electrolytes.

It is predicted that the MBA molecular layer adsorbed on the zinc electrode can accelerate the desolvation process and provide a large number of nucleation sites, thereby achieving dense zinc deposition. The wettability of different electrolytes on Zn foil during cycling was investigated via a dynamic contact angle test. As shown in Figure 3A and Figure S10, the initial contact angles of the electrolyte with/without MBA are 79° and 91°, respectively. As the time is prolonged, the contact angles of both electrolytes gradually decline. Nevertheless, the contact angle for the electrolyte with MBA is consistently lower than that of the electrolyte without MBA. The hydrophilic and zincophilic functional groups of MBA molecules can adhere to the surface of Zn metal anode to form a stable molecular adsorption layer, which will diminish the surface free energy to enhance the wettability of ZnSO_4_/MBA electrolyte. Thereby, the distribution of charge becomes more uniform, and the deposition kinetics are consequently improved. Figure 3B,C presents the differential charge density distribution of the optimized adsorption configurations for two models (H_2_O or MBA molecule on Zn surface). Evidently, a noticeable transfer of apparent charge can be observed between the Zn anode and the MBA molecule, suggesting a robust absorption interaction. It is noteworthy that the electron cloud overlaps between Zn atoms with O atoms of the ─C═O group and N atoms of the ─NH group for MBA molecule‐Zn (002) [26], which echoes the previous calculated adsorption energy results. It is well known that the nucleation process is critical for uniform Zn deposition. To elucidate the impact of the MBA additive on the nucleation process, the CV profiles of Zn//Cu asymmetric cells are presented in Figure 3D. Importantly, an increased nucleation overpotential is achieved for ZnSO_4_/MBA electrolyte compared with pure ZnSO_4_ electrolyte, which allows the formation of finer nuclei of deposited Zn, consequently preventing the random growth of dendrites. Furthermore, the random two‐dimensional diffusion of zinc ions or adsorbed Zn atoms on the surface of the electrode can result in the aggregation of Zn deposits, and this aggregation should be mitigated to ensure dendrite‐free deposition. Thus, the chronoamperometry (CA) curves were collected to reflect the diffusion behaviors of zinc ions or adsorbed zinc atoms on the electrode surface at a fixed potential of −150 mV. As demonstrated in Figure 3E, a continuously increasing trend in current can be observed for the Zn plating process in pure ZnSO_4_ electrolyte, indicating an expansion of the effective surface area, which could cause the formation of irregular Zn deposits on the Zn anodes. In sharp contrast, the variation in current for the Zn deposition process in the ZnSO_4_/MBA electrolyte is negligible after 50 s, suggesting that the random diffusion behavior of Zn^2+^ ions is suppressed. Furthermore, in situ optical microscopy was utilized to visually demonstrate the regulated Zn deposition behavior facilitated by the MBA additive. After just 5 min, Zn deposits started to emerge on the surface and cross‐section of the Zn plate in the ZnSO_4_ electrolyte, triggering a self‐amplifying process as electrochemical plating proceeded (Figure 3F). After half an hour, the surface and cross‐section of the electrode were blanketed with a layer of mossy Zn dendrites, resulting in an extremely rough surface texture. In contrast, throughout the electroplating process in the MBA‐contained electrolyte, the Zn anode consistently maintained a flat and dense surface morphology. SEM images (Figure 3G) reveal that the Zn surface cycled in the blank ZnSO_4_ electrolyte became extensively covered with irregular, flake‐like dendrite clusters and hexagonal by‐products, indicating unregulated Zn deposition and continuous side reactions between Zn and the aqueous electrolyte. With ZnSO_4_/MBA electrolyte, the Zn anode exhibits a flat and homogeneous morphology, indicating a well‐controlled deposition process (Figure 3H). These results are consistent with results from optical surface profilometry images of cycled Zn electrodes in electrolytes with/without MBA. A rough surface could be found on the Zn electrode without MBA additive, while the anode cycled in ZnSO_4_/MBA electrolyte displays a relatively uniform and compact surface with much lower surface roughness (Figure 3I,J). This behavior can be ascribed to the molecular adsorption layer of MBA on the Zn metal anode, which significantly accelerates the de‐solvation rate and captures the H_2_O molecules on the anode surface, thereby significantly suppressing zinc dendrites and side reactions.

### 2.4 Electrochemical Performance of Zn Anodes Using Different Electrolytes

**FIGURE 4:**
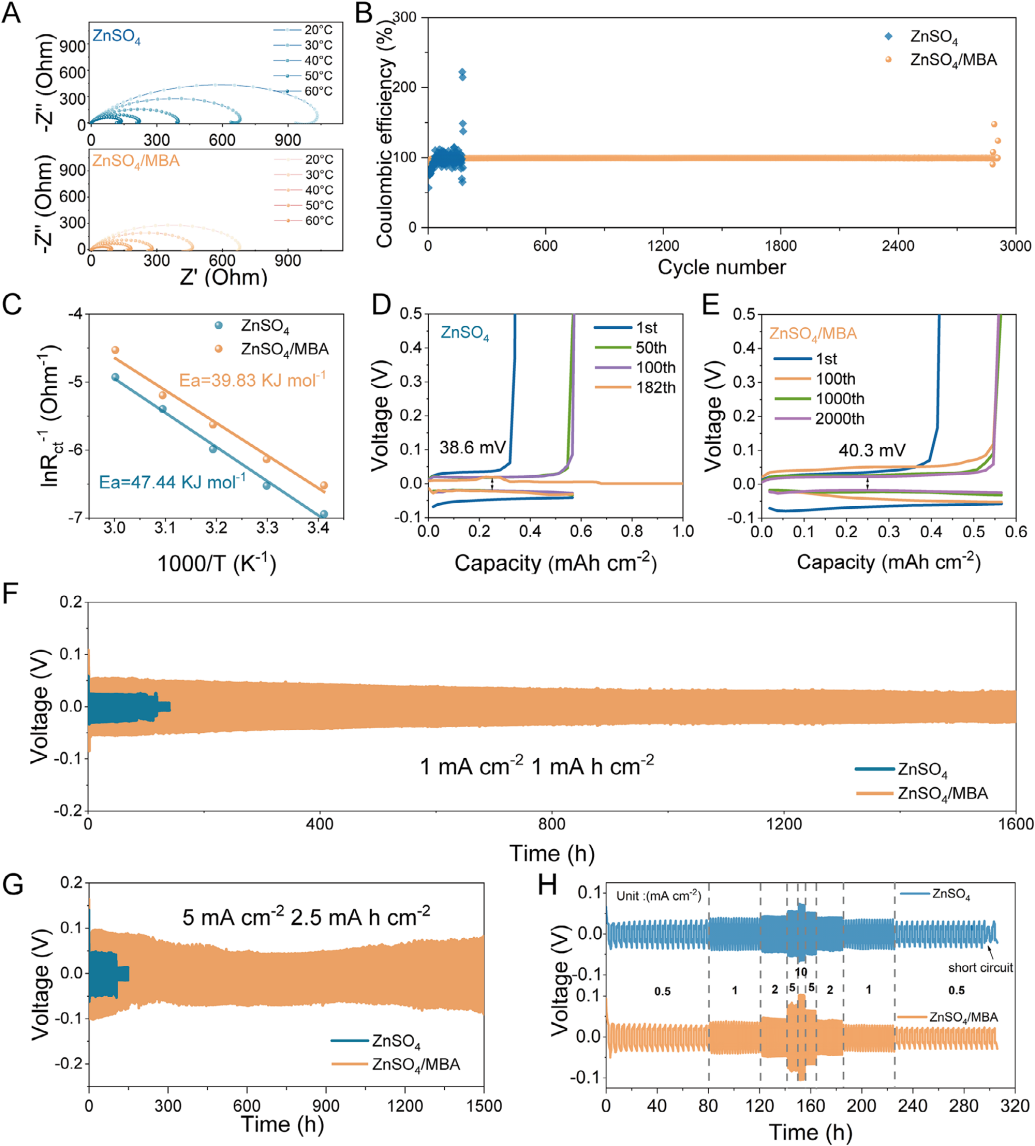
(A) Nyquist plots for the symmetric cells with ZnSO_4_ and ZnSO_4_/MBA electrolyte in the range of 20°C to 60°C. (B) Coulombic efficiency of Zn//Cu cells using various electrolytes. (C) Arrhenius curves and corresponding activation energies. The selected charge/discharge curves of Zn/Cu cells in (D) the ZnSO_4_ and (E) the ZnSO_4_/MBA electrolytes. Cycling performance comparison of symmetric cells with different electrolytes at (F) 1 mA cm^−2^ and 1 mAh cm^−2^ and (G) 5 mA cm^−2^ and 2.5 mAh cm^−2^. (H) Rate capabilities of the batteries using different electrolytes at a fixed capacity of 1 mAh cm^−2^.

To elucidate the impact of MBA on the transfer kinetics of zinc ions at the interface, the activation energy (*E*_a_) of Zn^2+^ is assessed through the Nyquist plots of symmetrical cells at ≈20°C–60°C (Figure 4A, C). After fitting with the Arrhenius equation, the *E*_a_ of the Zn anode in the electrolyte with MBA (39.83 kJ mol^−1^) is significantly lower than that in the ZnSO_4_ electrolyte (47.44 kJ mol^−1^). This suggests that the addition of MBA can reduce the diffusion energy barriers of Zn^2+^ to promote its migration rate at the interface. The CE is an important indicator for assessing the reversibility of Zn deposition/dissolution at the EEI. As presented in Figure 4B, the Zn//Cu asymmetric cell with the ZnSO_4_/MBA electrolyte shows stable cycling during 2800 cycles with a high CE of 99.74% under the condition of 1 mA cm^−2^ and 0.5 mA h cm^−2^. By contrast, the CE of the cell in the blank electrolyte fluctuates dramatically after short 200 cycles. The corresponding voltage profiles are depicted in Figure 4D,E, respectively. The cell with ZnSO_4_/MBA electrolyte displays smooth and stable voltage profiles throughout 2800 cycles. The significant improvement in reversibility and cycling stability can be ascribed to the inhibition of HER, which reduces the formation of irreversible Zn as a passivation byproduct [27]. The stabilizing effect of the MBA additive on the zinc anode is evaluated by galvanostatic charge/discharge test in symmetric cells. As illustrated in Figure 4F, the symmetric cell using blank electrolyte displays a cycling life of only 117 h at 1 mA cm^−2^. With the assistance of MBA additive, the stability of the symmetric cell is significantly enhanced with an extensive cycling duration of 1600 h, a nearly 14 times increase in the cycling lifespan. Notably, even under more stringent cycling conditions (5 mA cm^−2^ with 2.5 mAh cm^−2^), the cell utilizing ZnSO_4_/MBA electrolyte achieves a remarkable lifespan of 1500 h, significantly outperforming the cell utilizing ZnSO_4_ electrolyte (Figure 4G). Furthermore, the Zn deposition on the Zn anode surface maintains a dense and flat morphology without obvious dendrites or by‐products after 50 cycles using ZnSO_4_/MBA electrolyte (Figure S11). On the contrary, uncontrollable dendrite growth is observed on the surface of the zinc anode in the blank electrolyte, which could lead to the premature failure of symmetrical cells. The evaluation of rate capabilities for symmetric cells is shown in Figure 4H. Throughout testing at varying current densities, once the current density reverts to 0.5 mA cm^−2^, the cell with ZnSO_4_ electrolyte shows sign of degradation. As for the ZnSO_4_/MBA electrolyte, the improved interfacial transfer kinetics endows the Zn anode with effectively homogenized Zn^2+^ flow to realize an enhanced and more consistent rate performance.

### 2.5 Electrochemical Performance of the Full Cells

**FIGURE 5:**
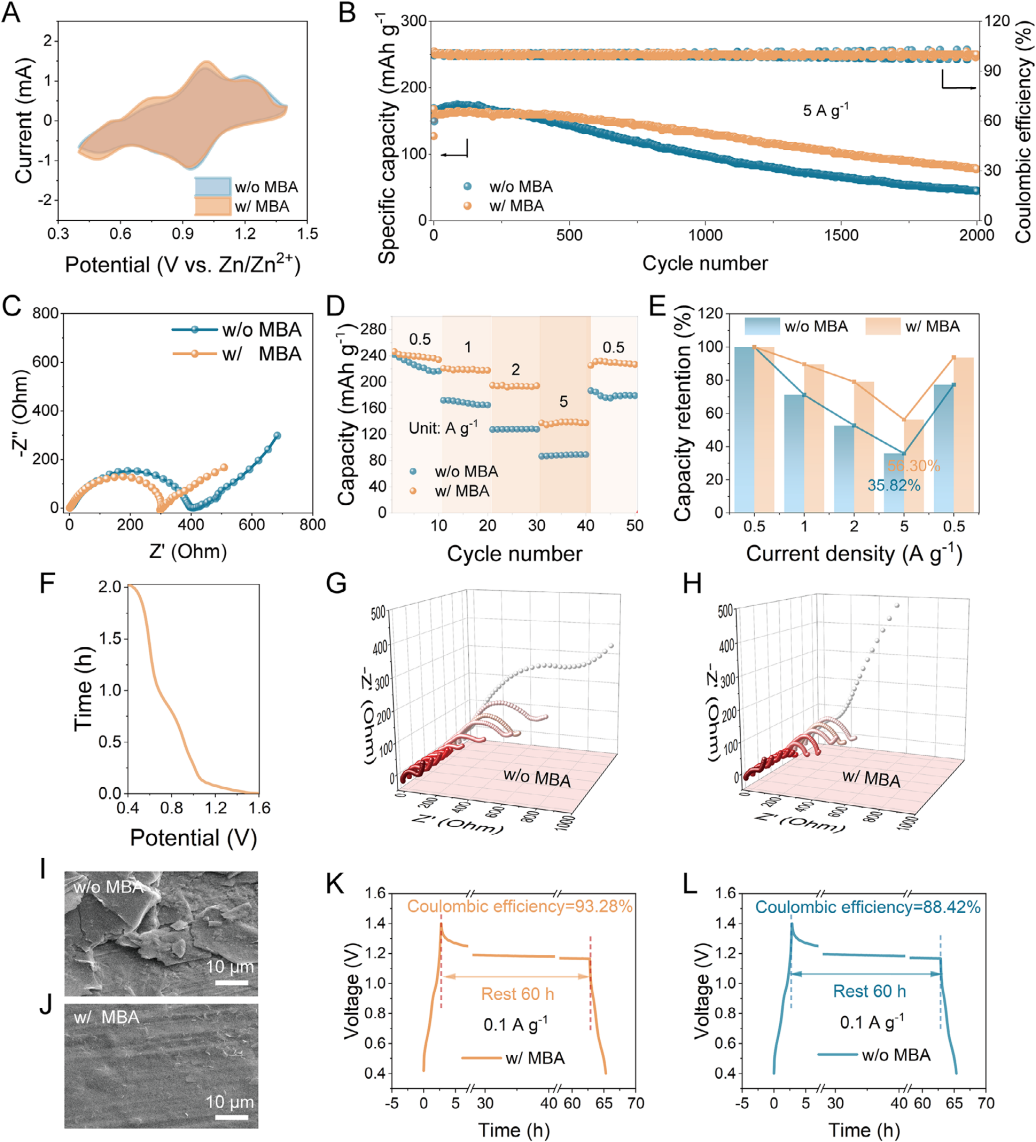
Electrochemical performances of full cells with different electrolytes. (A) CV curves at a scan rate of 1 mV s^−1^. (B) Long‐term cycling stability of Zn//V_2_O_5_ full cells at 5 A g^−1^ using electrolytes with/without MBA. (C) EIS curves of full cells before battery cycling. (D) Rate capabilities from 0.5 to 5 A g^−1^ and (E) corresponding capacity retention at different current densities. In situ EIS measurement of full cells: (F) discharge profile. EIS plots of (G) full cell with the ZnSO_4_ electrolyte and (H) the ZnSO_4_/MBA electrolyte. (I,J) SEM images of Zn anodes of full cells in various electrolytes after 30 cycles. Self‐discharge curves of full cells (K) without and (L) with MBA.

To validate the viability of MBA additives in practical applications, a V_2_O_5_ cathode was selected to assemble Zn//V_2_O_5_ full cells for testing their electrochemical performance, with the zinc trifluoromethanesulfonate (Zn(OTf)_2_) as the electrolyte due to its better compatibility with V_2_O_5_ (Figure S12). As illustrated in Figure 5A, no additional peaks are observed in the CV curves of Zn//V_2_O_5_ cells using two distinct electrolytes at 1 mV s^−1^, indicating that the addition of MBA will not trigger extra reactions [28]. In Figure 5B, the long cycling performance of full cells was evaluated at 5 A g^−1^. Impressively, the initial discharge capacity of the Zn//V_2_O_5_ cell with MBA additive is 168.03 mAh g^−1^, and the capacity still maintains at 77.7 mAh g^−1^ after 2000 prolonged cycles. The capacity of the full cell using the pristine electrolyte decreases dramatically to 44.4 mAh g^−1^, with rapid capacity fading and a lower capacity retention rate (26.2%). In addition, as the inhibition of passivation side reactions could effectively promote the rapid reaction kinetics, the full cell with MBA exhibits the lower charge transfer resistance (Figure 5C). Subsequently, the rate performance was tested over a wide current density range from 0.5 to 5 A g^−1^ (Figure 5D), and the capacity retention at the corresponding currents was shown in Figure 5E. At various current densities, the full cell with MBA additive consistently exhibits higher specific capacities compared to the cell using pristine electrolyte. The capacity of the cell containing MBA remains at 56.3% of that at 0.5 A g^−1^, even at the high current density of 5 A g^−1^. Furthermore, when the current density is restored from 5 to 0.5 A g^−1^, the capacity returns to the initial level of approaching 250 mAh g^−1^. This suggests the enhanced rate performance enabled by the MBA additive. In situ electrochemical impedance spectroscopy (EIS) was conducted for the first discharge process of the Zn//V_2_O_5_ full cell from 1.4 to 0.4 V, and the corresponding voltage profile was displayed in Figure 5F. The charge transfer resistance in the EIS spectra of the full cell using MBA maintains a relatively small value throughout the discharge process, and its variation is also smaller than that of the full cell using blank electrolyte (Figure 5G,H). This indicates the accelerated charge transfer kinetics at the interface. Figure 5I,J displays the SEM images of the Zn anode surface in the full cells with two different electrolytes after 30 cycles at 5 A g^−1^. It is noteworthy that the full cell using the MBA additive exhibits a notably smooth surface without accumulation of passivation by‐products. According to Figure 5K,L, the full cell with MBA additive achieves a high CE of 93.28%, surpassing that of the full cell without MBA (88.42%). This affirms that the addition of an MBA can substantially mitigate side reactions at the interface and enhance the cycling lifespan [29]. The above outstanding electrochemical properties delivered by the full cells further highlight the pivotal role of the addition of MBA in securing high reversibility in capacities, alleviating passivation, and extending lifespan.

## 3 Conclusion

In conclusion, the incorporation of the MBA additive has led to the creation of an innovative aqueous electrolyte that optimizes hydrogen‐bonding networks within water molecules, thereby enhancing the durability of the Zn anode during cycling. Unlike the commonly reported electrolyte additives, which can only serve as hydrogen bond acceptors, the MBA additive simultaneously acts as both a hydrogen bond donor and acceptor due to its unique amide group, including ─C═O and ─NH functional groups. This double hydrogen bonding interactions between MBA and H_2_O molecules effectively break the inherent hydrogen‐bonding structure of H_2_O molecules within the aqueous electrolyte, significantly diminishing the reactivity of water. In addition, trace MBA molecules with polar groups tend to adsorb on the zinc metal electrode and capture active water molecules in the EDL, thus accelerating the de‐solvation process of Zn^2+^ and inhibiting side reactions caused by water decomposition. The Zn//Cu asymmetric cells with MBA‐contained electrolyte achieve a high CE of 99.74% with a stable lifespan of 2800 cycles at 1 mA cm^−2^. In addition, the ZnSO_4_/MBA electrolyte endows the Zn//Zn symmetric cells with an ultra‐long cycle life of 1500 h at 5 mA cm^−2^ and 2.5 mAh cm^−2^. More encouragingly, the addition of the MBA additive can also improve the cycling stability of Zn//V_2_O_5_ full cells with high‐capacity reversibility and a long lifespan of 2000 cycles. These findings highlight the critical role of regulating hydrogen bonding in developing the low‐cost aqueous electrolytes, which are essential for the practical application of aqueous zinc metal batteries with extended lifespan.

## 4 Experimental Section

### 4.1 Preparation of Electrolytes

The base electrolyte for the symmetric cell and the asymmetric cell, a 2 m solution of ZnSO_4_, is prepared by dissolving zinc sulfate heptahydrate (ZnSO_4_·7H_2_O, Aladdin, AR, 28.756 g, 2 m) in deionized water (37.4 mL). Subsequently, *N*, *N*'‐methylenebisacrylamide (C_7_H_10_N_2_O_2_, named as MBA, Aladdin, AR) was introduced to the prepared 2 m ZnSO_4_ solution (20 mL) at different concentrations (0.046, 0.092, and 0.139 g represents15, 30, and 45 mm, respectively) to obtain MBA/ZnSO_4_ electrolytes. The most effective concentration of MBA was identified to be 30 mm. The full cell performance testing and characterization were conducted using a 2 m zinc trifluoromethanesulfonate (Zn(OTf)_2_) solution as the baseline electrolyte, with 30 mm MBA‐containing (Zn(OTf)_2_) electrolyte as the experimental group.

### 4.2 Preparation of Electrode

The zinc foil of 0.1 mm thickness (12 mm diameter) is used as the anode. The cathode was prepared by drying a slurry consisting of vanadium pentoxide (V_2_O_5_, Aladdin, AR), carbon black, and polyvinylidene fluoride, which were blended in *N*‐methyl‐2‐pyrrolidone (AR, >99.0 %) at a weight ratio of 7:2:1. This mixture was uniformly coated onto Ti foil (0.1 mm thick) and then dried in a vacuum oven at a temperature of 60°C for 12 h. The V_2_O_5_ mass loading was about 1.2 mg cm^−2^.

### 4.3 Material Characterization

^1^H NMR (BRUKER‐AVANCE III HD 500 MHz) and FTIR (Nicolet Summit X) were used to analyze the disruption and reconstruction of hydrogen bond networks by MBA. Raman spectroscopy of different electrolytes was done with Rainshaw PLUS. XPS of the Zn surface was conducted using Thermo SCIENTIFIC Nexsa. Passivation by‐product formation on Zn anode surface was investigated by XRD (D8 ADVANCE, BRUKER) in the scanning range between 5°and 80°. The wettability of various electrolytes on the Zn anode surface was observed through contact angle (Shanghai Fangrui, JCY‐1). The surface morphology of the Zn electrode in different electrolytes was characterized by SEM (ZEISS SUPRA55). The surface state of the zinc anode in two distinct electrolytes after cycling was carried out using an optical surface profilometer (WYKONT9100).

### 4.4 Electrochemical Measurement

All CR2025‐type coin cells were assembled using glass fiber (GF/D, Whatman) to act as the separator. The asymmetric battery configuration was established using copper foil as the working electrode and zinc foil to function as both the counter electrode. Galvanostatic current charge/discharge tests with different electrolytes were carried out using the NEWARE battery‐testing instrument (CT‐4008Tn‐5V50mA). A comprehensive set of electrochemical analyses, encompassing linear scan voltammetry (LSV), cyclic voltammetry (CV), Tafel, chronoamperometry (CA), and EIS (from 100 kHz to 0.1 Hz), was conducted using an AutoLab electrochemical workstation (PGSTAT302N). LSV and CV were conducted using a three‐electrode setup, featuring a Zn sheet as the working electrode, a platinum (Pt) counter electrode, and an Ag/AgCl reference electrode. The CA measurements applied a constant overpotential of −150 mV. The EDLC was evaluated through CV curves of Zn//Zn symmetric cells in a voltage range of −15 to 15 mV under various scanning rates from 2 to 10 mV s^−1^. The Zn//V_2_O_5_ full cells were cycled within a potential window of 0.4–1.4 V.

### 4.5 Theoretical Calculations

All structural optimizations and detailed analysis were carried out using Gaussian 09 software [30]. The hybrid B3LYP functional with Grimme's D3 dispersion [31] correction was combined with a 6–311G (d, p) basis set for these molecular calculations. The basis set superposition error correction was also included in the binding energy calculations for molecular structures. All periodic calculations were conducted at the DFT level using the projector augmented wave potential [32]. The Vienna ab initio simulation package [33] was employed for this purpose. The generalized gradient approximation parameterized by Perdew–Burke–Ernzerhof (PBE) functional [34] was used in combination with a plane‐wave basis set of 500 eV kinetic energy cut‐off, with dispersion contributions included within Grimme's D3 scheme [31]. A force convergence threshold of 0.05 eV Å^−1^ and the threshold of self‐consistent field calculations of 1 × 10^−4^ eV were consistently used during all periodic calculations.

## Conflicts of Interest

The authors declare no conflicts of interest. Prof. Chade Lv is a member of the Exploration editorial board, and he was not involved in the handling or peer review process of this manuscript.

## Supporting information




**Supporting Information file 1**: exp270076‐sup‐0001‐SuppMat.docx

## Data Availability

The data that support the findings of this study are available from the corresponding author upon reasonable request.
