# Supplementary material for: Disrupting Hydrogen Bond Network Connectivity With a Double‐Site Additive for Long‐Life Aqueous Zinc Metal Batteries
Source: Exploration (Beijing). 2025 Aug 21;5(5):20240007. doi: 10.1002/EXP.20240007 (PMC12561446; doi:10.1002/EXP.20240007)
Supplement: Supplementary file 1 — Supporting Information file 1: exp270076‐sup‐0001‐SuppMat.docx [file EXP2-5-20240007-s001.docx]

**Supporting Information for**

**Disrupting hydrogen bond network connectivity with a double-site additive for long-life aqueous zinc metal batteries**

Dongping Chen^1,2,6^, Xipo Ma^1,2,6^, Weihao Xv^1,2,6^, Chunshuang Yan^1,2*^, Pengbo Lyu^3*^, Qiang Zhu^4^, Huaming Yu^1^, Zhenren Gao^5^, Chade Lv ^1,2*^

^1^State Key Laboratory of Space Power-Sources, School of Chemistry and Chemical Engineering, Harbin Institute of Technology, Harbin 150001, PR China

^2^MIlT Key Laboratory of Critical Materials Technology for New Energy Conversion and Storage, School of Chemistry and Chemical Engineering, Harbin Institute of Technology, Harbin 150001, PR China

^3^Hunan Provincial Key Laboratory of Thin Film Materials and Devices, school of Material Sciences and Engineering, Xiangtan University, Xiangtan, 411105, PR China

^4^Institute of Materials Research and Engineering, A*STAR, 2 Fusionopolis Way, Innovis, #08-03, 138634 Singapore

^5^Hunan Provincial Key Laboratory of Micro-nano Energy Materials and Application Technologies, College of Physics and Electronic Engineering, Hengyang Normal University, Hengyang 421002, PR China

^6^These authors contributed equally to this work.

**Results and Discussion**


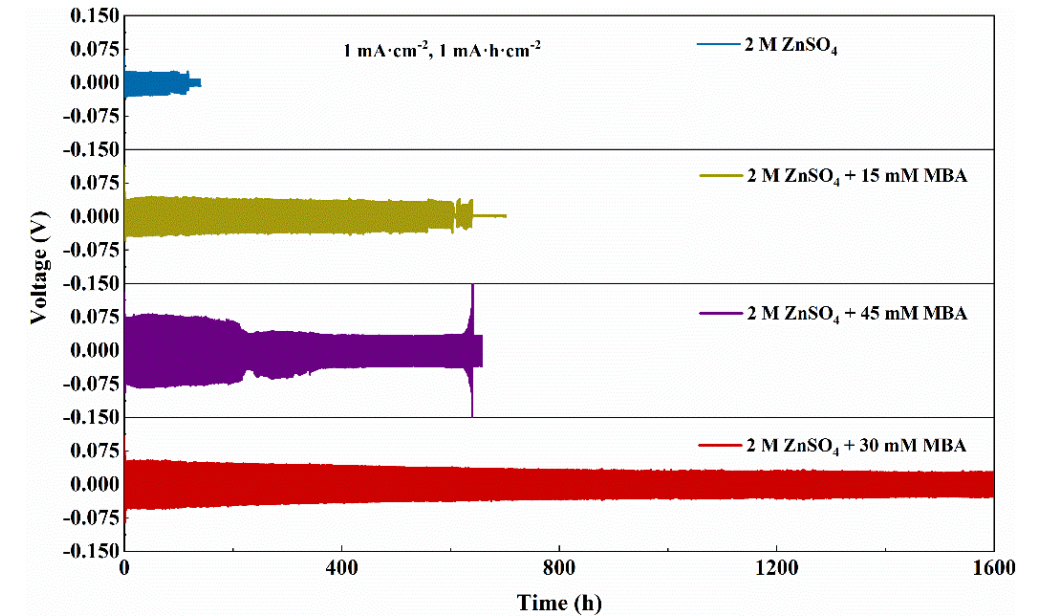


**Figure S1** The comparison of galvanostatic charge and discharge measurement of MBA with different concentrations in Zn//Zn symmetric cells at 1 mA cm^-2^ and 1 mAh cm^-2^.


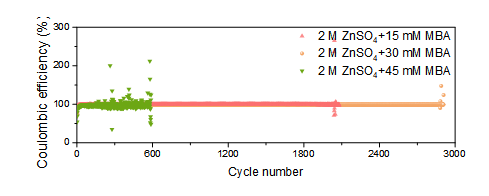


**Figure S2** The comparison of Coulombic efficiency of MBA with different concentrations in in Zn//Cu asymmetric cells at 1 mA cm^-2^ and 0.5 mAh cm^-2^.


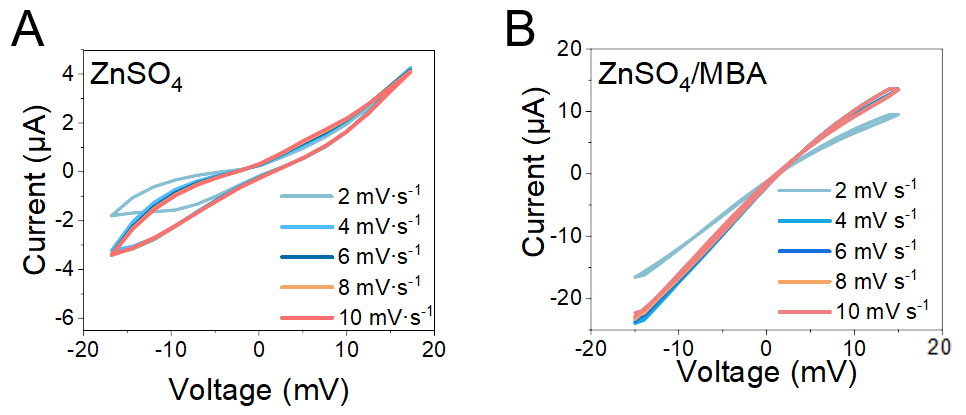


**Figure S3** CV curves of Zn||Zn cells in a voltage range of -15 mV to 15 mV under various scanning rates in (A) pure ZnSO_4_ and (B) ZnSO_4_/MBA electrolytes.


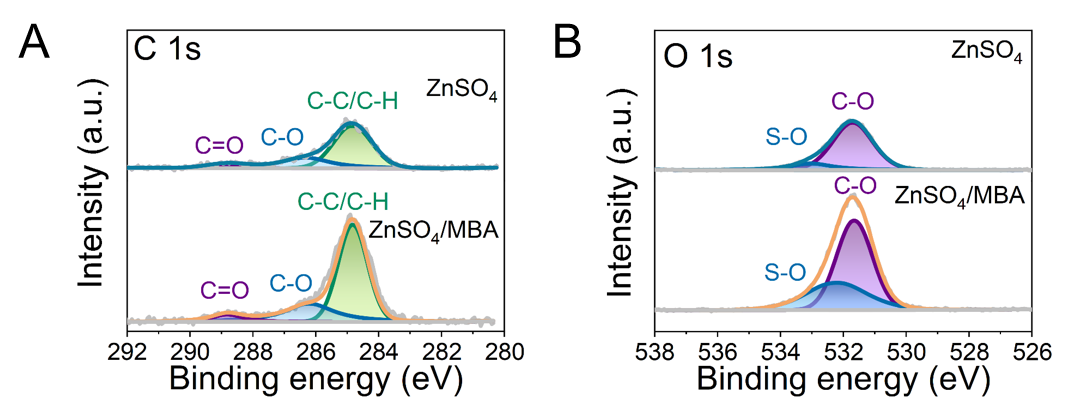


**Figure S4** (A) C 1s and (B) O 1s XPS spectra of the Zn anode after 50 cycles in ZnSO_4_ and ZnSO_4_/MBA electrolyte.


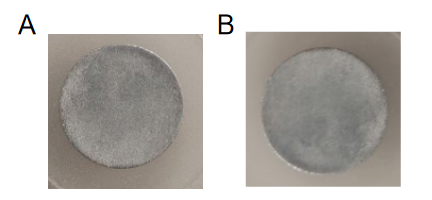


**Figure S5** Optical photographs of Zn foil soaked in electrolyte (A) without and (B) with MBA additives for 7 days.


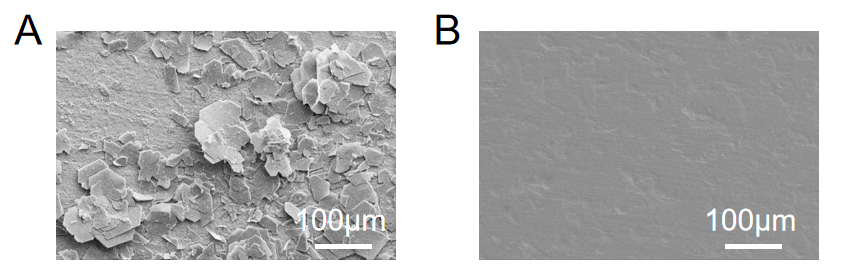


**Figure S6** SEM images of Zn foil soaked in electrolyte (A) without and (B) with MBA additives for 7 days.


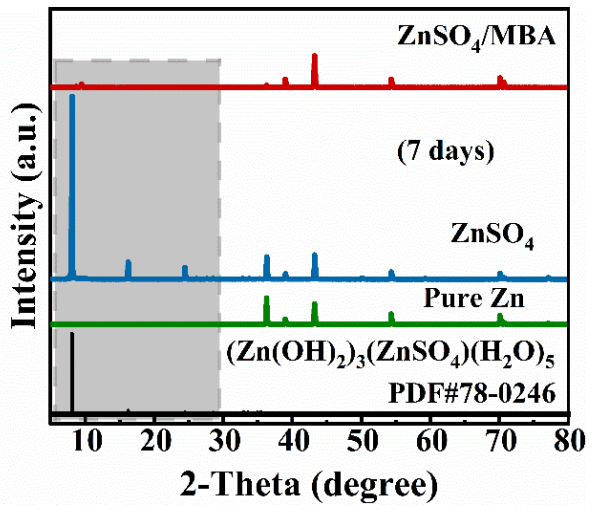


**Figure S7** XRD patterns of fresh Zn and Zn anode immersed in different electrolytes after 7 days.


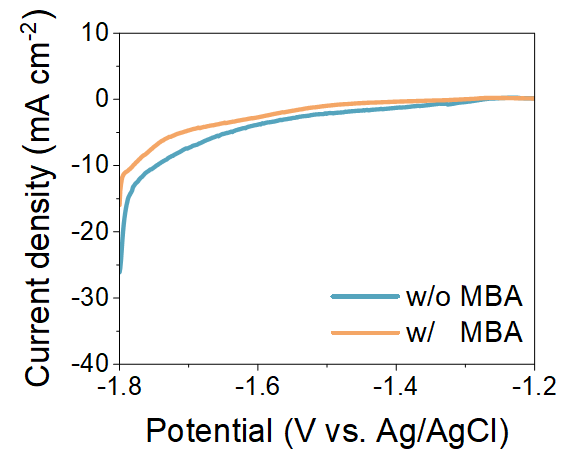


**Figure S8** Linear scanning voltammetry of different electrolytes indicating hydrogen evolution reaction.


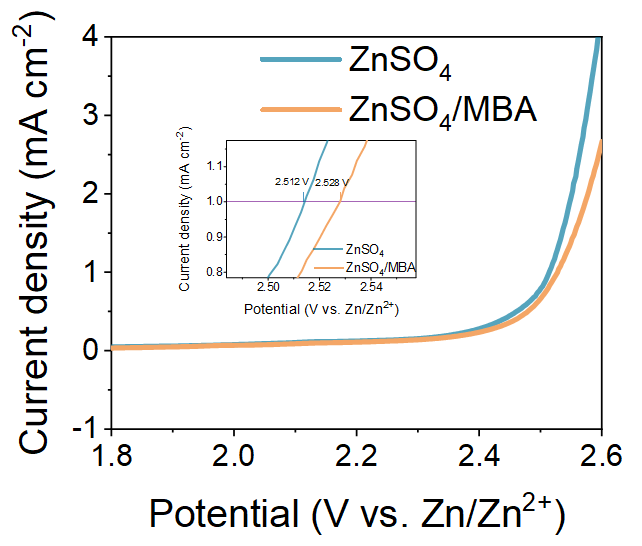


**Figure S9** Linear scanning voltammetry of different electrolytes indicating oxygen evolution reaction.


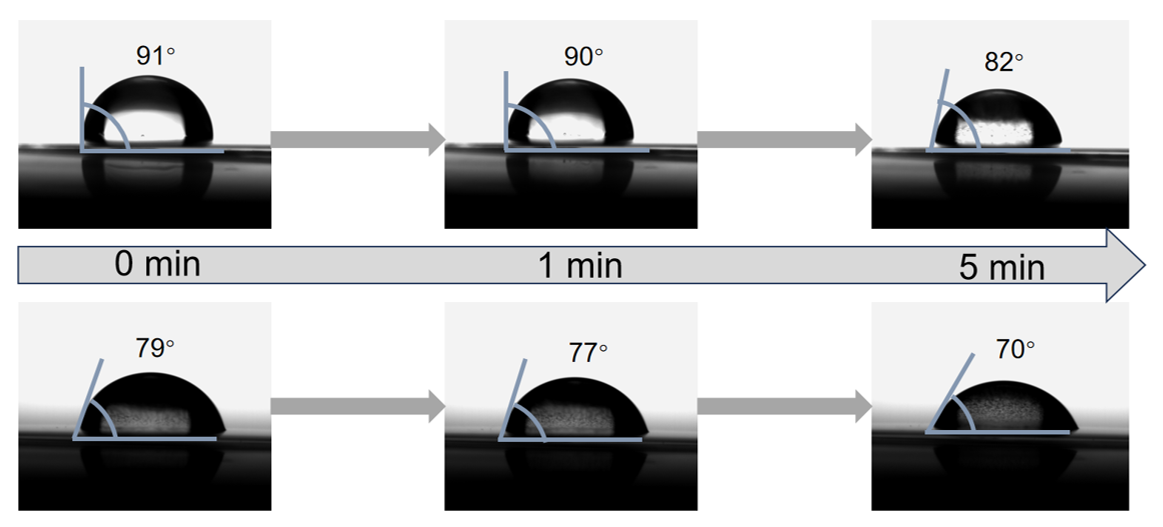


**Figure S10** Dynamic contact angles of different electrolytes on zinc metal surfaces.


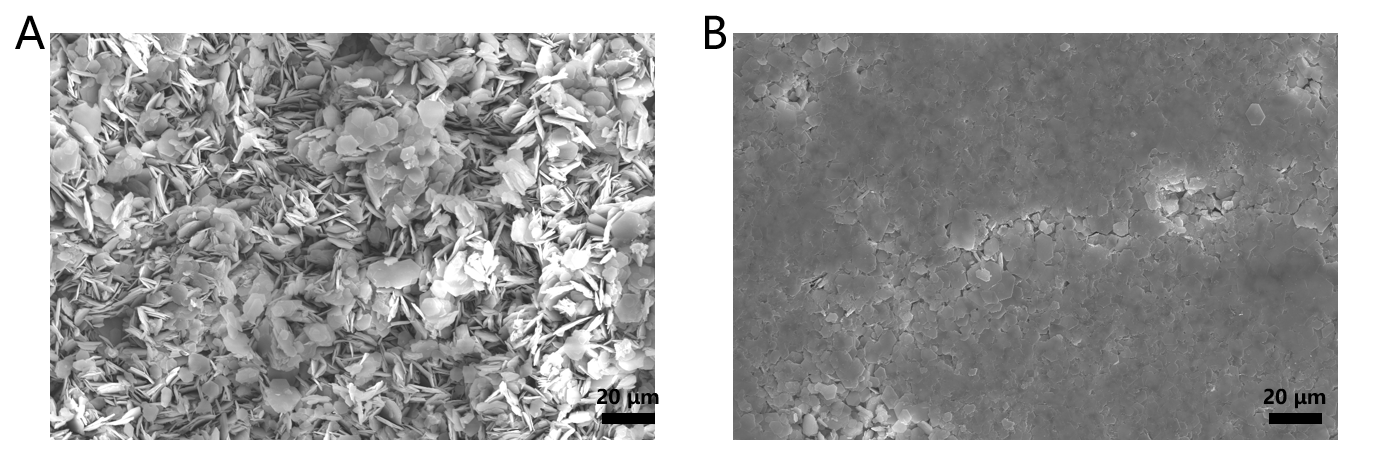


**Figure S11** SEM images of Zn anode after 50 cycles at 1 mA cm^-2^ and 1 mAh cm^-2^ in the electrolyte (A) without MBA and (B) with MBA.


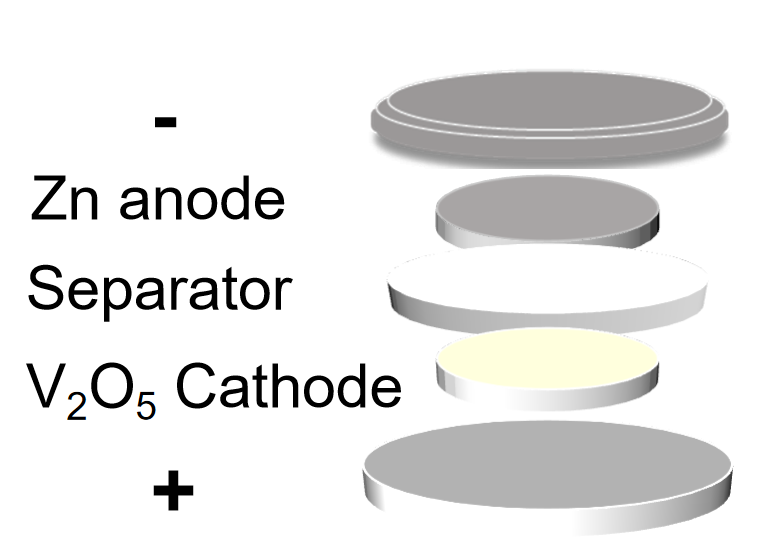


**Figure S12** Schematic illustration of Zn//V_2_O_5_ full cell.
